# Progress of Palladium Nanomaterials for Tumor Diagnosis and Therapy

**DOI:** 10.1021/acsomega.5c08165

**Published:** 2025-12-18

**Authors:** Zhi Li, Wanli Yang, Shujiang Wang, Changyi Ma, Jia Sha, Xuan Meng, Kai Ding

**Affiliations:** † Department of General Surgery & Research Institute of General Surgery, Jinling Hospital, Affiliated Hospital of Medical School, Nanjing University, 305 Zhongshan East Road, Xuanwu District, Nanjing City, Jiangsu Province 210002, China; ‡ Department of Digestive Surgery, Xijing Hospital, Air Force Medical University, No. 127, West Changle Road, Xincheng District, Xi’an City, Shaanxi Province 710032, China; § State Key Laboratory of Holistic Integrative Management of Gastrointestinal Cancers and National Clinical Research Center for Digestive Diseases, Xijing Hospital of Digestive Diseases, Air Force Medical University, No. 127, West Changle Road, Xincheng District, Xi’an City, Shaanxi Province 710032, China; ∥ First Detached Outpatient Department, Jinling Hospital, Affiliated Hospital of the Medical School, Nanjing University, 305 Zhongshan East Road, Xuanwu District, Nanjing City, Jiangsu Province 210002, China; ⊥ Department of Orthopaedics, Jinling Hospital, Affiliated Hospital of Medical School, Nanjing University, 305 Zhongshan East Road, Xuanwu District, Nanjing City, Jiangsu Province 210002, China; # Hepatobiliary Surgery Department, National Cancer Centre/National Clinical Research Centre for Cancer/Cancer Hospital, Chinese Academy of Medical Sciences and Peking Union Medical College, No. 17, Panjiayuan South Lane, Chaoyang District, Beijing 100021, China

## Abstract

Tumors are the most serious threats to global health. While conventional treatment methods, such as chemotherapy and radiotherapy, often result in harsh side effects due to poor targeting, palladium-based nanomaterials (Pd-NMs) offer new possibilities for improved treatment outcomes. With exceptional photothermal conversion, a long circulation half-life, high tumor accumulation, favorable optical/magnetic properties, and great biocompatibility, Pd-NMs may reshape the diagnosis and treatment of tumors. Building upon these potential benefits, Pd-NMs play critical roles in diagnosis by serving as imaging agents, tumor cell detectors, and biological probes. In treatment, most Pd-NMs are administered intravenously, allowing for passive tumor targeting through the effect of enhanced permeability and retention (EPR), which inhibits cancer cell proliferation and migration. Their small size reduces the impact on normal tissue and adverse reactions, and they are widely used in photothermal therapy, photodynamic therapy, chemotherapy, radiotherapy, chemodynamic therapy, and combined therapy. However, the Pd-NMs could interfere with immune cell functions and induce oxidative stress. Prolonged exposure to Pd-NMs can lead to organ damage, including the kidneys, lungs, and liver. They are mainly excreted via the kidneys, with clearance affected by size, surface modification, and exposure routes. Looking ahead, clinical applications need to overcome challenges (e.g., improved tumor targeting, long-term biosafety, cost reduction, and large-scale production). Consequently, future research should focus on improving efficacy, developing new strategies, and exploring long-term biosafety to promote clinical translation. This review aims to support related studies.

## Graphical abstract

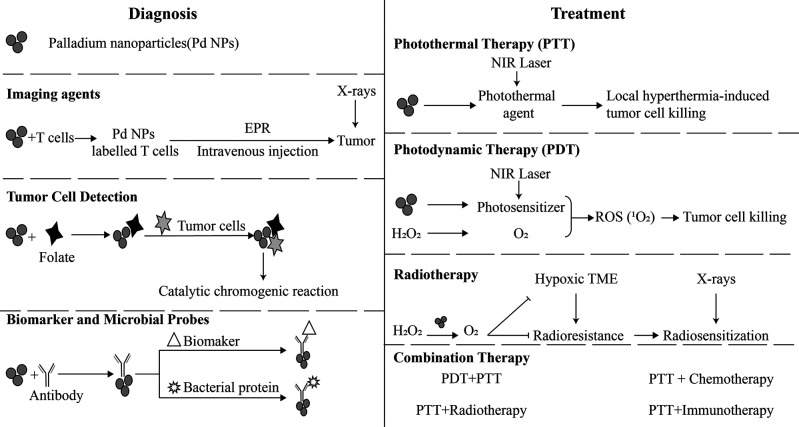

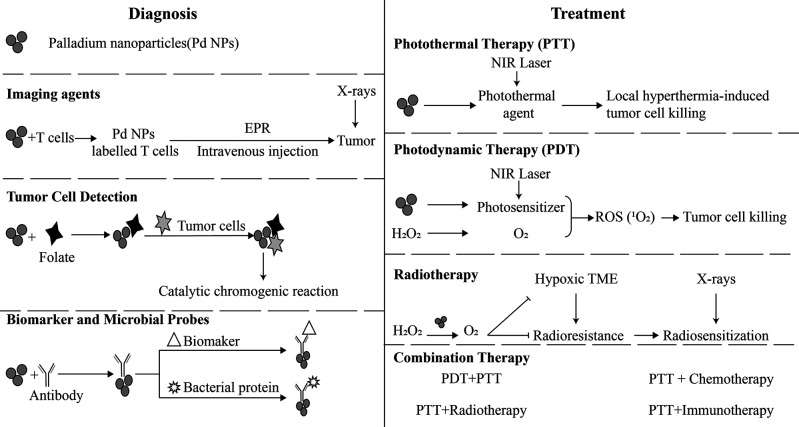

## Introduction

Tumor poses a significant public health and economic threat to contemporary society, accounting for 16.8% of global deaths.^1^ Although chemotherapy and radiotherapy remain mainstream treatments, their nonspecific targeting often leads to severe side effects (such as organ toxicity, bone and marrow suppression) and drug resistance issues. Building on these challenges, growing evidence proves that nanotechnology is redefining cancer theranostics through engineered nanomaterials capable of simultaneous diagnosis and treatment.^2^ Recently, palladium-based nanomaterials (Pd-NMs) have garnered attention for their great photothermal conversion, which is driven by their ultrathin two-dimensional (2D) structures (such as Pd nanosheets) and crystallinity-dependent plasmonic effects. These attributes position them as promising candidates for next-generation near-infrared (NIR) activated therapies, though scalability and batch-to-batch consistency remain practical hurdles.^3^

Since Huang et al. synthesized palladium nanosheets (Pd-NSs) in 2011, their uniform hexagonal morphology (5–120 nm) and NIR absorption properties have made them an ideal material for photothermal therapy (PTT).^4^ Due to the unique optical properties of Pd-NSs, notably the light absorption peak primarily located in the NIR region, they have become a promising material for PTT applications. Then, the utilization of Pd-NMs has sparked widespread research interest, emerging as a highly dynamic and intensively explored area.^5^

Building upon this foundation, recently, Pd-NMs have presented excellent potential in the diagnosis and treatment of tumors. They can serve not only as contrast agents for tumor imaging^13^ but also as biological probes for detecting tumor markers,^14^ aiding physicians in earlier and more accurate tumor diagnosis. In addition, Pd-NMs can be applied in PTT,^15^ photodynamic therapy (PDT),^15^ chemodynamic therapy (CDT),^16^ radiotherapy,^17^ chemotherapy,^18^ sonodynamic therapy (SDT),^19^ and piezoelectric catalytic therapy^20^ for tumors. To enhance the stability of Pd-NMs, improve their uptake efficiency in tumor cells, researchers have constructed various composite structures, such as palladium nanoparticles (Pd NPs)^6^ (Figure 1A), Pd-NSs (Figure 1B),^7^ palladium amphiphile triglycerol monostearates nanoparticles (Pd@TGMs NPs) (Figure 1C),^8^ Pd/H-TiO2 ^9^ (Figure 1D), Au/PdNSs: Au­(core)/Pd­(shell) nanospheres (Au: Gold; Pd: Palladium)^10^ (Figure 1E), Au/PdNSs/TiO2: Au­(core)/Pd­(shell) nanospheres coated with TiO2 shell^10^ (Figure 1F), polyethylene glycol-Coated Pd nanosheets (PEG-PdN)^11^ (Figure 1G) and Ternary metal–metalloid palladium–copper-boron alloy microporous nanospheres (PdCuB MNs)^12^ (Figure 1H). Furthermore, due to the complex pathogenesis of tumors, combination therapy strategies can help improve treatment efficacy. Synergistic effects with immunotherapy, radiotherapy, and other treatments enhance therapeutic outcomes, such as PTT combined with PDT.^6^

**Figure 1:**
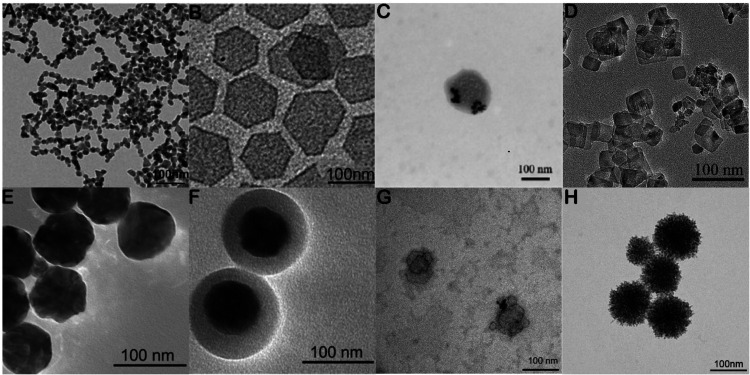
(A) TEM image of palladium nanoparticles (Pd NPs), adapted with permission from ref 6. Copyright 2023, the Royal Society of Chemistry. (B) TEM image of palladium nanosheets (Pd NSs), reprinted with permission from ref 7. Copyright 2020, American Chemical Society. (C) TEM image of palladium amphiphile triglycerol monostearates nanoparticles (Pd@TGMs NPs), reprinted with permission from ref 8. Copyright 2020, American Chemical Society. (D) TEM images of Pd/H-TiO2, adapted with permission from ref 9, Copyright 2022, BMC. (E) TEM image of Au/PdNSs: Au­(core)/Pd­(shell) nanospheres, adapted with permission from ref 10 (Au: Gold; Pd: Palladium). Copyright 2022, Springer Nature. (F) TEM image of Au/PdNSs/TiO2: Au­(core)/Pd­(shell) nanospheres coated with TiO2 shell, adapted with permission from ref 10. Copyright 2022, Springer Nature. (G) TEM image of polyethylene glycol-coated Pd nanosheets (PEG-PdN), adapted with permission from ref 11. Copyright 2024, MDPI. (H) Ternary metal–metalloid palladium–copper-boron alloy microporous nanospheres (PdCuB MNs), adapted with permission from ref 12. Copyright 2023, BMC. Scale bar, 100 nm.

Taken together, these findings set the stage for this review, which introduces the role of Pd-NMs in tumor diagnosis and treatment. Then we discuss recent research advances in Pd-NMs for tumor diagnosis and treatment. We also explored various combined treatment strategies involving Pd-NMs for tumors. Then, we analyze the immunogenicity, toxicology, and metabolic clearance mechanisms of Pd-NMs. Finally, we discuss the potential challenges that future research may face. It is hoped that this review can provide insights into the role of Pd-NMs in tumor diagnosis and treatment, and offer assistance for subsequent Pd-NMs-related research.

## Applications of Pd-NMs in Tumor Diagnosis

Pd-NMs have excellent biocompatibility, prolonged circulation half-life, high tumor accumulation, and favorable optical and magnetic properties. In terms of tumor diagnosis, the excellent properties of palladium nanomaterials demonstrate potential application value in imaging agents, tumor cell detectors, and marker probes, among other aspects. The applications of Pd-NMs in tumor diagnosis are outlined in Table 1.

**Table 1:** Palladium Nanomaterials in Tumor Diagnosis^a^

type of diagnosis	materials	refs
XFI imaging	PdNPs	^21^
CT imaging	Pd@Au-PEG	^13^
MRI imaging	Pd nanosheets labeled with Gd or radionuclides	^22^
tumor cell detection	PdNPs/CMC-COF-LZU1	^23^
tumor cell detection	PdNPs/COP	^24^
CTC detection (MCF-7)	nanomaterials and palladium–iridium cubic nanozymes	^26^
detection of HER2	Pd(−0.1 V)/CNTs	^31^
detection of PSA	Pd TPs	^28^
detection of CA242	rGO-Au-Pd nanocomposite	^14^
detection of miRNA	SERS-based LFA biosensor with CHA	^30^
detection of CEA	Cu_2_S with Pd NPs	^33^
detection of PD-L1^+^ exosomes	PdCuBMNs and Au@CuCl_2_ NWs	^12^

a Abbreviation: XFI, X-ray fluorescence imaging; CT, computed tomography; MRI, magnetic resonance imaging; PdNPs, palladium nanoparticles; CNTs, carbon nanotubes; PSA, prostate specific antigen; CA-242, carbohydrate antigen 242;HER2, human epidermal growth factor receptor 2; CTC, circulating tumor cells; Pd-NCs, palladium nanoclusters; rGO-Au-Pd, reduced graphene oxide-gold–palladium; Pd TPs, palladium triangular plates; CEA, Carcinoembryonic antigen; Cu_2_S, cuprous sulfide; Programmed cell death ligand 1 protein-positive, PD-L1^+^; PdCuBMNs, palladium-copper-boron alloy microporous nanospheres; NWs, nanowires.

### Imaging Agents

Recent studies showed that Pd-NMs can be used as suitable agents for tumor imaging. They have been applied in X-ray fluorescence imaging (XFI),^21^ computed tomography (CT),^13^ and magnetic resonance imaging (MRI).^22^

XFI is a method that uses the glow emitted when X-rays interact with materials to identify the elements present. This method has significantly improved the detection of tumors. Although not a conventional direct diagnostic tool for tumors, XFI can provide physicians with vital information about the elemental composition within tumors, thereby assisting in more precise diagnoses. Previous research has indicated that cytotoxic T cells infiltrating tumor tissue can serve as a parameter for early assessment and prediction of treatment responses, which is essential for noninvasive, sensitive, and longitudinal imaging. Kahl et al. found that the palladium imaging agent used in XFI can track CD8^+^ T cells, and through estimation, when Pd is used as an imaging agent to monitor related T cells, the labeling efficiency required in conventional scenarios is only 0.009–0.004 pg Pd per T cell, while for subcutaneous targets, it is 0.029–0.013 pg Pd per T cell, which is significantly lower than the corresponding requirements when Au is used as an imaging agent (0.286–0.044 pg Au per T cell in conventional scenarios and 0.945–0.133 pg Au per T cell for subcutaneous targets). This study suggests that Pd is a potentially valuable XFI imaging agent, warranting further exploration.^21^

The CT scan is a common imaging method that visualizes the internal structures of the human body by utilizing the differential absorption of X-rays. Using the seed growth method, Chen et al. synthesized Pd@Au-PEG with hexagonal palladium nanosheets as the core, modified by thiol polyethylene glycol. These nanosheets possess strong near-infrared absorption capacity, excellent stability, and a long in vivo blood circulation time. Pd@Au-PEG has significant advantages as a contrast agent for photoacoustic imaging and CT. Its high atomic number components (Pd/Au) give it X-ray absorption capacity surpassing that of iodine agents, which can effectively reduce the dosage of contrast agents. In addition, tumor-specific accumulation is achieved through passive targeting mediated by the enhanced permeability and retention (EPR) effect, significantly improving the imaging signal-to-noise ratio. Pd@Au-PEG displays performance superior to traditional contrast agents in CT imaging, providing a new strategy for precise tumor diagnosis.^13^

In the field of tumor diagnosis, Pd-NMs have also demonstrated significant advantages in the design of MRI contrast agents owing to their unique physicochemical properties. The ultrasmall palladium fluoride nanosheets (such as FDP-Pds) developed by Guo et al. act as ^19^F MRI contrast agents, and their mechanism is in sharp contrast to that of traditional contrast agents. Compared with traditional gadolinium-based ^1^H MRI contrast agents, ^19^F MRI has a significantly improved signal-to-noise ratio (SNR) due to the natural scarcity of ^19^F signals in the body (no endogenous background), which can provide more accurate quantitative imaging and deep tissue penetration capabilities. Although gadolinium agents are widely used in T1-weighted imaging, they are susceptible to physiological background interference, have a low signal-to-noise ratio, and carry potential toxicity risks. In addition, the ultrasmall size (<10 nm) of Pd-NMs ensures excellent biocompatibility and renal clearance ability, avoiding the problem of long-term retention of gadolinium agents in the liver and spleen. Surface functionalization (such as polyethylene glycol modification) of Pd-NMs can integrate Gd3^+^ to enhance ^1^H MRI signals while maintaining the high specificity of ^19^F MRI, enabling the synergistic application of multimodal imaging (such as SPECT/MRI/PAI). In summary, FDP-Pds possess ultrahigh ^19^F signal intensity, EPR-mediated tumor-targeted enrichment, and multifunctional integration capabilities, suggesting that FDP-Pds are MRI contrast agents with potential clinical application value.^22^

### Tumor Cell Detection

The detection of tumor cells is helpful for the early screening and diagnosis of tumors, and can also be used to evaluate the efficacy of tumor treatment and detect tumor recurrence. Sensitive and accurate recognition of tumor cells in clinical fluids facilitates the diagnosis of patients with tumors. Pd NPs in situ grown on carboxymethyl cellulose-modified covalent organic framework hydrogels (PdNPs/CMC-COF-LZU1) can utilize their catalytic performance for specific chemical reactions to achieve high sensitivity and accurate detection of cervical cancer cells (HeLa). This detector could sensitively discover cancer cells from serum samples, and the detection limit of this detector is 100 cells/mL (Figure 2A,B).^23^ Photoelectrochemical (PEC) sensors with high sensitivity and outstanding selectivity may offer a new approach to cell sensing. A cathodic PEC cell sensor integrated with covalent organic Polymers (COP) and Pd NPs can detect tumor cells by catalyzing the oxidation of dopamine and using aminochrome derivatives as electron acceptors, providing a new powerful tool for early tumor diagnosis. The detection limit of this PEC cytosensor is 8 cells/mL, which is better than that of the PdNPs/CMC-COF-LZU1 detector constructed by Sun et al. (100 cells/mL). Besides, the large dynamic detection limit of PEC ranges from 10 to 10^6^ cells per mL. Furthermore, this PEC cytosensor offers several advantages, including high selectivity, excellent reproducibility, and outstanding stability, and can be used to directly detect many cancer cells via integration with corresponding specific aptamers.^23,24^

**Figure 2:**
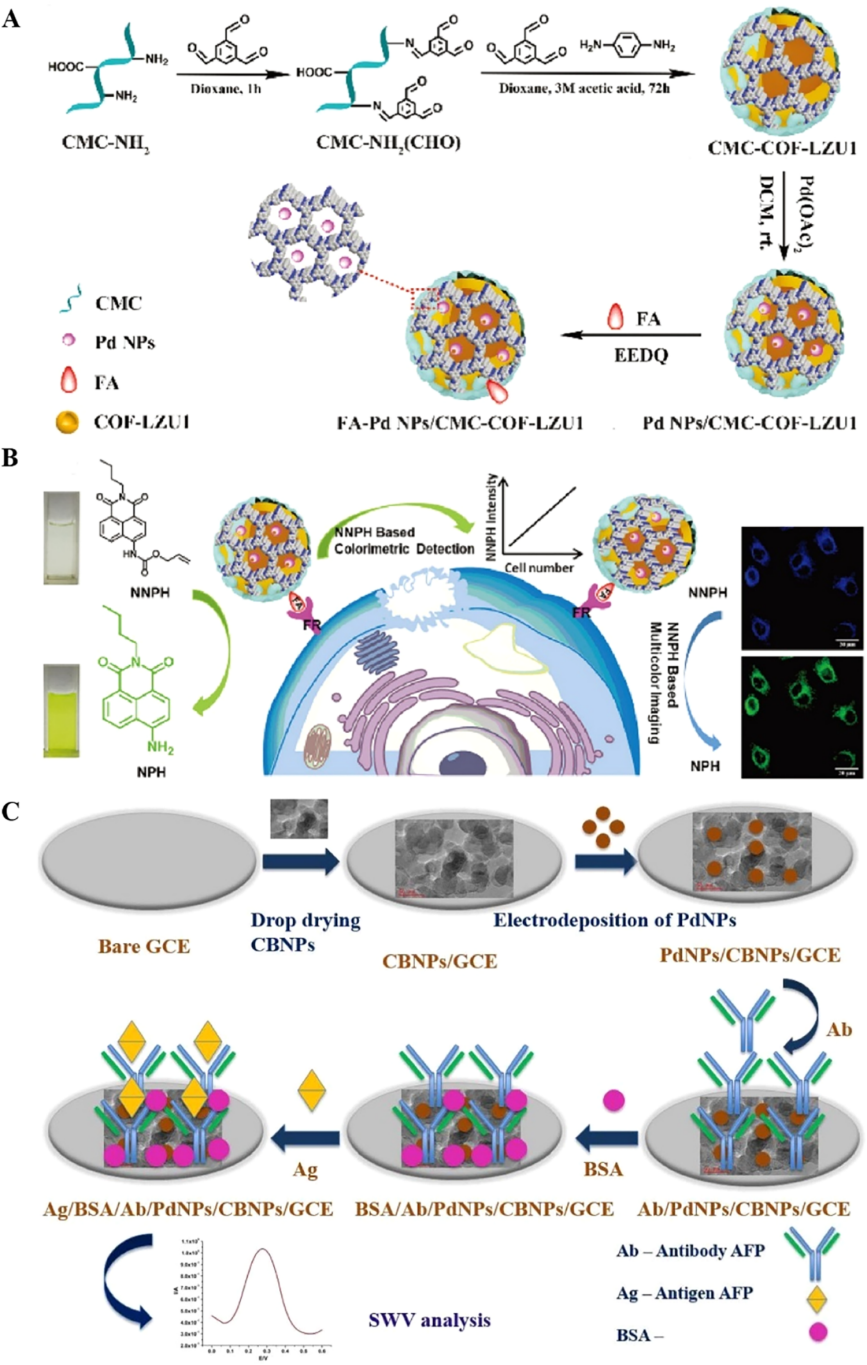
(A) Illustration of the synthesis process of FA-Pd NPs/CMC-COF-LZU1, adapted with permission from ref 23. Copyright 2020,the Royal Society of Chemistry. (B) Schematic illustration of the dual functions of FA-Pd NPs/CMC-COF-LZU1 for cancer cell imaging, adapted with permission from ref 23. Copyright 2020, the Royal Society of Chemistry. (C) Schematic diagram for the fabrication process of the hybrid CBNPs/PdNPs immunosensor for the detection of a cancer biomarker: α-feto protein (AFP), adapted with permission from ref 27. Copyright 2023, the Royal Society of Chemistry.

Coordination complexes as cell imaging probes have great potential. Saini et al. developed an anthracene-conjugated fluorescent palladium­(II) cyclometalated probe [PdL (1) Cl], which stains the cell membrane and uses confocal and fluorescence lifetime imaging microscopy, providing an important tool for the study of cell membrane heterogeneity and the understanding of biological systems, especially for the membrane staining of tumor and normal cells.^25^ Fu et al. developed an electrochemical sensing model based on nanomaterials and palladium–iridium cubic nanozymes, which achieved high-sensitivity, high-specificity detection of CTCs by catalyzing hydrogen peroxide (H_2_O_2_) to amplify the signal. This immunosensor has a detection limit of 2 MCF-7 cells per mL, offering a new and efficient method for early tumor diagnosis and prognosis monitoring.^26^ And this immunosensor achieves the best detection limit, which is lower than the cell detector constructed by Cui et.al (8 cells/mL)^24^ and Sun et al. (100 cells/mL).^23^

### Biomarker and Microbial Probes

Recent studies showed that Pd-NMs were designed as probes to recognize specific tumor biomarkers such as programmed cell death ligand 1 (PD-L1), α-fetoprotein (AFP), prostate-specific antigen (PSA), carcinoembryonic antigen (CEA), carbohydrate antigen 24–2 (CA-242), human epidermal growth factor receptor 2 (HER2), and microRNAs (miRNAs), thereby achieving highly sensitive tumor detection. PD-L1-positive exosomes represent promising biomarkers for cancer diagnosis. Chang et al. create an electrochemical aptamer detector based on Au@CuCl_2_ nanowires and trimetallic palladium-copper-boron alloy microporous nanospheres to enable high-precision identification of PD-L1­(+) exosomes from human serum samples. The sensor maintained good linearity within an extensive concentration range of 6 orders of magnitude. It achieved a low detection limit of 36 particles/mL, providing an effective device for identifying NSCLC patients in the early stage.^12^ To achieve early cancer screening and diagnosis, Olorundare et al. developed an electrochemical immunosensor using a hybrid nanocomposite of carbon black nanoparticles (CBNPs) and PdNPs as the platform (Figure 2C). This platform targets a tumor-associated antigen, AFP, as the detection biomarker. With linear detection ranges of 0.005–1000 ng mL^–1^, the low detection limits of AFP under square wave voltammetry (SWV) and electrochemical impedance spectroscopy (EIS) modes were 0.0039 ng mL^–1^ and 0.0131 ng mL^–1^, respectively. The immunosensor exhibits excellent performance, along with good stability, reproducibility, and selectivity. Its practical application potential has been verified through detection in the human serum matrix.^27^ PSA is a seminal plasma protein secreted by prostate epithelial cells. PSA is generated in both normal prostate cells and prostate cancer cells. Still, its levels are significantly upregulated in prostate cancer patients, making it a standard tumor marker for prostate cancer. Researchers developed an ultrasensitive PSA aptamer sensor based on the “turn-on–off-turn-on” model, utilizing fluorescence resonance energy transmission between g-C3N4 quantum dots and palladium nanoplates, which offered an efficient tool for the early diagnosis of prostate cancer. Besides, this aptamer sensor can accurately measure PSA, with a concentration range linearly distributed from 10 picograms per milliliter to 50 nanograms per milliliter.^28^ CEA is a vital tumor biomarker for patients with colorectal cancer. Ratiometric electrochemiluminescence (ECL) sensors can eliminate environmental interference and achieve accurate detection. Shang et al. created a dual-potential ratiometric ECL sensor derived from single luminophore and coreactant, using palladium nanoclusters (Pd-NCs) prepared with a DNA duplex as a template, thereby achieving sensitive detection of CEA, with a detection limit of 87.1 ag/mL (S/N = 3) and a linear range from 100 ag/mL to 10 ng/mL, providing new technical support for early cancer diagnosis.^29^ CA-242 is a common diagnostic biomarker for detecting pancreatic cancer and colorectal cancer. Du et al.^14^ constructed a novel label-free electrochemical immunosensor using reduced graphene oxide-gold–palladium (rGO-Au-Pd) nanocomposite materials. It showed high sensitivity and specificity for CA-242 detection, with a linear detection range of 0.001–10,000 U mL^–1^ and a detection limit of 1.54 × 10^–3^ U mL^–1^. Recently, microRNA (miRNA) detection has attracted significant attention for early cancer detection and diagnosis. Li and colleagues developed a lateral flow analysis (LFA) biosensor based on surface-enhanced Raman scattering (SERS) and a catalytic hairpin assembly (CHA) amplification strategy. This biosensor enables highly sensitive and specific identification of miR-196b and miR-106b, which are correlated to laryngeal squamous cell carcinoma (LSCC). The detection limits for miR-106b and miR-196b were as low as 23.17 and 46.94 aM (attomolar, 1 aM = 10–18 mol/L) in PBS. It could also be used to quantify the target in serum from patients with LSCC.^30^ Metal nanostructures are considered attractive candidates for designing new biosensors due to their large surface area, accelerated kinetics, and better affinity. Joshi et al. used a one-step electro-reduction method to load palladium nanostructures onto oxidized carbon nanotubes, thereby creating a label-free electrochemical immunosensor suitable for the sensitive detection of HER2. The sensor can produce an ultrasensitive electrochemical response to HER2 (detection limit: 1 ng/mL), with a detection range of 10 to 100 ng/mL, showing high sensitivity and promising potential in screening HER2-positive breast cancer patients.^31^ After a comprehensive analysis of these biosensors, most sensors exhibit high sensitivity for detecting biomarkers and can detect them at quite low concentrations. Due to the different measurement units for the biomarkers, comparing their detection limits was difficult. Most biomarkers are sourced from serum, which contains many different proteins and components. Thus, the specificity of the biomarker detector should also be investigated. Besides, when applying the biomarker detector in the clinical diagnosis process, the economic cost should also be considered. Further studies are needed to improve the clinical application value of Pd-NMs in the detection of tumor biomarkers. *Helicobacter pylori* (*H. pylori*) colonization in the human stomach increases the risk of gastritis, ulcers, and gastric cancer. BabA is an outer membrane protein and one of the primary virulence factors of *H. pylori*. A BabA-based immunosensor,^32^ hybridized with nanomaterials and loaded with Pd NPs and reduced graphene oxide/poly­(3,4-ethylenedioxythiophene) on an Au electrode through electrodeposition, has achieved highly sensitive, specific, and reproducible detection of *H. pylori*. The linear detection range of this immunosensor for detecting the *H. pylori* was 0.2–20 ng/mL. This immunosensor provides a new tool for monitoring *H. pylori*, aiding early screening of populations at high risk of gastric cancer.^32^

## Applications of Pd-NMs in Tumor Therapy

Most Pd-NMs enter the human body via intravenous injection or other routes of administration. The EPR effect is a phenomenon driven by the unique vascular structure of tumors. These Pd-NMs exploit the EPR effect to achieve passive targeting and accumulation at tumor sites. Subsequently, Pd-NMs inhibit the proliferation and migration of cancer cells. Importantly, the small dimensions of Pd-NMs reduce their impact on normal tissues following administration, resulting in relatively mild adverse reactions. To date, these Pd-NMs have been widely applied in various tumor therapies, including PTT, PDT, chemotherapy, radiotherapy, chemodynamic therapy, and combination therapy, with specific uses outlined in Table 2.

**Table 2:** Applications of Pd-NMs in Tumor Therapy^a^

type of therapy	materials	refs
PTT	MnO_2_@Pd@PPy/GO	^15^
PTT	Pd-TAT	^34^
PTT	Pd NPs-TMC	^35^
PTT	Dap-Pd NFs	^36^
PTT	PdNC and PdCeO_2_	^37^
PTT	Pd@Au	^13^
PPTT	Pd-Ncap	^38^
PDT	H–Pd–NSs	^7^
PDT	PdRu-RCE@PCMNPs	^42^
PDT	PdH(0.2)-Ir@RBT	^39^
CDT	GOx@Pd@ZIF-8	^16^
radiotherapy	PdTe	^44^
radiotherapy	FePd@CNTs	^45^
radiotherapy	PdPt NPs	^43^
radiotherapy	folate-coupled Pd NPs	^46^
chemotherapy	CaCO_3_@Pd@C	^52^
chemotherapy	Au@PdNDs	^18^
chemotherapy	PdPL-TCZ	^48^
chemotherapy	M13@Pd/NLG	^70^
chemotherapy	rGO-MWCNTs-AgL(2)/PdL(2)	^49^
chemotherapy	Res–PdNPs	^50^
sonodynamic therapy	PdCu(x)@LDH	^19^
magnetic hyperthermia therapy	Pd/Fe-oxide NPs	^56^
piezoelectric catalytic therapy	Pd-BTO	^20^
bioorthogonal catalysis therapy	Pd NPs (Pd@UiO-66)	^57^
combination therapy	RCL@Pd@CuZ	^68^
combination therapy	Pd-NSs/CpG-ODNs	^67^
combination therapy	Pd/DOX@hydrogel	^6^
combination therapy	PdH@MnO_2_·Ce6@HA	^62^
combination therapy	HBPdC	^72^
combination therapy	EcN–QS–iLux@Pd@MC540	^60^
combination therapy	PdNPs-Exo and 5-FU	^69^
combination therapy	RMNP (containing palladium-103)	^66^

a Abbreviation: polypyrrole/graphene oxide nanocomposite codecorated with manganese dioxide and Pd NPs, MnO_2_@Pd@PPy/GO; Pd-NSs modified with cell-penetrating peptides (TAT: CGGYGRKKRRQRRR), Pd-NSs-TAT; trimethyl chitosan-coated, Pd NPs-TMC; PEGylated Pd@Au nanoplates, Pd@Au-PEG; porous Pd NPs combined with paclitaxel, Pd NPs-PTX; trimethyl chitosan-coated Pd NPs, Pd NPs-TMC; Photothermal Therapy, PTT; Chemodynamic Therapy, CDT; Plasmonic Photothermal Therapy, PPTT; Photodynamic Therapy, PDT; exosomes isolated from PdNPs-induced macrophages, PdNPs-Exo; resveratrol-functionalized palladium nanoparticles, Res–PdNPs.

### Photothermal Therapy (PTT)

PTT is an emerging treatment scheme that injects materials with efficient photothermal conversion ability into the body. Then, external light sources (such as NIR) are utilized to irradiate the material to convert light energy into heat energy in the tumor tissue, thereby inhibiting tumor growth and killing cancer cells. Since nanoparticles can regulate the absorption of exogenous energy locally to produce heat, they use high-temperature conditions to kill local tumor cells while minimizing thermal damage to surrounding healthy cells. Noble metal nanoparticles (NP) have inherent properties such as antiangiogenesis, antibacterial, and anti-inflammatory effects. Due to their unique characteristics, including slowing down the migration and invasion of cancer cells, nanoparticles are widely used clinically. Moreover, owing to their surface plasmon resonance effect, Pd-NMs have good NIR absorption and photothermal conversion efficiency, making them ideal materials for tumor PTT.

Wu et al. synthesized a polypyrrole/graphene oxide (MnO_2_@Pd@PPy/GO) nanocomposite codecorated with manganese dioxide and Pd NPs, which showed brilliant photothermal conversion performance (achieved about 50 °C after 10 min of light exposure), pH-responsive enzymatic catalytic activity, and greater MRI performance. Cell experiments demonstrated that using the nanocomposites as theranostic nanoagents enabled MRI-guided combined cancer treatment. The combined approach reduced cancer cell viability to 30%, compared to 91% with PTT alone and 74.7% with CDT alone. This provides a new nanotreatment agent for MRI-guided cancer PTT and CDT, opening up novel avenues for controllable synthesis and utilizing multicomponent nanomaterials.^15^ Tumor metastasis is an important reason for the failure of tumor therapy. Inhibiting metastasis is a key step in improving patient survival rates. Gao et al. constructed a cell-penetrating peptide TAT (CGGYGRKKRRQRRR) modified Pd NSs (abbreviated as Pd-TAT) strategy,which not only significantly inhibits the growth of primary cancer cells, but also inhibits the cancer cell migration and invasion, improving the therapeutic effectiveness of PTT.^34^ Besides, Bangde et al. found that trimethyl-chitosan (TMC)-coated Pd NPs showed great potential as 808 nm NIR laser-mediated PTT agents for breast cancer. Compared to uncoated Pd NPs, TMC-coated Pd NPs have great biocompatibility and physiological stability.^35^ He et al. found that daptomycin micelle-stabilized palladium nanoflowers (Dap-PdNFs) could rapidly increase the temperature from 26.8 to 52.0 °C within 10 min under irradiation of 808 nm NIR, with a photothermal conversion efficiency of up to 38%. Moreover, when the cancer cells were treated with Dap-PdNFs in the absence of 808 nm NIR laser, the viability of the cancer cells exceeded 95%, demonstrating the excellent biocompatibility of Dap-PdNFs. With its excellent biocompatibility and high photothermal conversion efficiency, Dap-PdNFs offer an efficient and safe approach for PTT.^36^ In the study by Cruz et al., hydrogenated nanofluids composed of PdHx nanoparticles exhibited notable temperature increases of over 30 °C after 3 min of irradiation with a diode laser (829.1 nm). In comparison, nanofluids with PdCeO2H nanoparticles exhibited a temperature increase of approximately 11 °C under identical conditions. Overall, these palladium–hydrogenated nanofluids demonstrated significant temperature increases under laser irradiation, suggesting their effectiveness as PTT agents for cancer treatment.^37^ Recent studies have found that the “plasmonic effect” enhances photothermal efficiency, and plasmonic photothermal therapy (PPTT), as a special PTT treatment strategy, has gradually become the preferred treatment method for various cancers. A novel bimetallic palladium nanocapsule (Pd-Ncap) constructed by Singh et al. achieved a high photothermal conversion of 49% at relatively low concentrations of nanoparticles and laser power densities, with the ability to scavenge reactive oxygen species (ROS). This Pd-Ncap provides an effective PTT treatment strategy for tumor patients.^38^

### Photodynamic Therapy (PDT)

PDT is a crucial cancer treatment strategy. PDT can effectively kill tumor cells and inhibit their proliferation by generating cytotoxic ROS. Molecular oxygen yields reactive oxygen species through energy excitation or electron transfer. Reactive oxygen species mainly include superoxide anion (O_2_^–•^), H_2_O_2_, hydroxyl radical (^•^OH), and singlet oxygen (^1^O_2_). Among them, ^1^O_2_ is the reactive oxygen species that plays a key therapeutic role in PDT, while other reactive oxygen species only play an auxiliary role. Photosensitizers could absorb light energy at specific wavelengths and initiate chemical reactions through energy transfer or electron transfer. Their core function is to convert light energy into chemical energy or biological effects, and they are widely used in PDT, photocatalysis, photopolymerization, and other fields. The ROS generation efficiency of photosensitizers is crucial to determining the efficacy of PDT and is therefore the focus of current research.

Currently, photosensitizers can be divided into three major categories according to material properties: organic, inorganic/metal-based, and nano/supramolecular systems. Their core commonality is having high intersystem crossing (ISC) efficiency and high singlet oxygen quantum yield (ΦΔ). Among them, nano/supramolecular system photosensitizers, through nanocarriers or supramolecular assembly, solve problems such as poor water solubility and insufficient targeting of traditional sensitizers, and are currently a cutting-edge development direction. At present, palladium-based PDT photosensitizers can be separated into 4 categories based on their core composition and functional design, following the logic from basic structure to intelligent integration. The first category is pure palladium/palladium alloy-based nanophotosensitizers, with pure palladium or palladium-based alloys as the core functional units, which have both excellent photodynamic performance and photothermal effects, and some have hypoxia tolerance. Among them, porous palladium nanosheets (H–Pd–NSs) have a tumor inhibition rate as high as 99.7% and can be used for image-guided hypoxic tumor therapy.^7^ Palladium–iridium alloy (Pd–Ir)-based materials^39^ can precisely control the reactive oxygen species generation threshold by adjusting the near-infrared laser wavelength, reducing damage to normal tissues. The second category is palladium-organic complex photosensitizers, constructed by the complexation of palladium ions with organic ligands (porphyrins, linear tetrapyrroles). They integrate the advantages of organic ligands in absorbing light in the therapeutic window and the strong intersystem crossing efficiency of metal ions, with excellent reactive oxygen species generation efficiency (Pd-Tripor is the highest in the series). They have good biocompatibility in the dark, and their therapeutic activity under light is significantly better than that of free-base porphyrins (the IC50 of Pd-Tripor is only 9.6 μM, lower than 18.2 μM of Tripor).^40^ New linear tetrapyrrole complexes also solve problems such as complex synthesis and purification, and insufficient biocompatibility of traditional macrocyclic tetrapyrroles.^41^ The third category is palladium-based composite/hybrid nanophotosensitizers, constructed by compounding palladium with other components (metals, polymers, carbon materials, inorganic nanomaterials). They can improve reactive oxygen species generation efficiency by separating photogenerated electron–hole pairs through the built-in electric field of cobalt oxide-palladium nanocube Schottky junctions,^41^ or integrate functions such as enzyme catalysis, photothermal conversion, and MRI like MnO_2_@Pd@PPy/GO (manganese dioxide-palladium-polypyrrole/graphene oxide composite materials), achieving MRI-guided synergistic therapy of PTT and CDT with both high efficiency and low side effects. The fourth category is palladium-based intelligent responsive nanophotosensitizers, which integrate response mechanisms (thermal response, light wavelength regulation) on the basis of palladium-based core functions, enabling precise control of the timing or amount of reactive oxygen species release. Among them, the thermally responsive nanoparticles PdRu-RCE@PCMNPs (palladium–ruthenium alloy-polypyrrole complex-thermally responsive phase change materials)^42^ have both tumor fluorescence/photothermal imaging and multimodal synergistic therapy capabilities, and can inhibit the growth and metastasis of breast tumors. The intelligent nanosystem PIH@R (PdH(0.2)-Ir@RBT) can avoid reactive oxygen species overflow through laser wavelength adjustment, maximizing the protection of normal tissues while efficiently eradicating tumors.

### Chemodynamic Therapy (CDT)

CDT is an emerging tumor treatment strategy. It mainly triggers chemical reactions by leveraging the tumor microenvironment (such as high-concentration H_2_O_2_, acidic pH, etc.) to generate cytotoxic ROS (such as ^•^OH), thereby inducing apoptosis of tumor cells while minimizing damage to normal tissues. The lack of H_2_O_2_ and ROS in cancer tissues pose a significant challenge to cancer CDT. Jin et al. developed an effective ROS producer, GOx@Pd@ZIF-8, which includes Pd nanozymes, glucose oxidase (GOx), and zeolite imidazole framework-8 (ZIF-8). This material promotes apoptosis of tumor cells by synergistically blocking glucose metabolism and enhancing ROS production.^16^

### Radiotherapy

Currently, palladium nanomaterial-mediated tumor radiotherapy mainly focuses on radiosensitization, regulation of the tumor microenvironment, or targeted delivery. The aim is to overcome radioresistance and improve radiotherapy efficacy. These therapies rely on the surface properties, stability, and radiosensitization potential of noble metal nanoparticles. Palladium-based materials, including bimetallic alloys, nanozymes, and composite carriers, act through multiple mechanisms. For example, bimetallic nanoalloys (PdAu, PdPt) enhance the radiation-induced killing of cancer cells via component synergy. Green, chemically synthesized PdAu composites significantly enhance the efficacy of proton therapy on colon cancer cells, with minimal toxicity to normal cells.^17^ PdPt nanoalloys show better X-ray/proton radiosensitization efficiency than other nanostructures. This highlights the key influence of nanocomponent ratios and structures on sensitization effects.^43^ The hypoxic TME is a core cause of radioresistance. To relieve hypoxia of TME and support enhanced radiotherapy efficacy, PdTe nanozymes catalyze the decomposition of intratumoral H_2_O_2_ to generate oxygen.^44^ FePd@CNTs integrate radiosensitization and biological barrier penetration, which significantly improves material accumulation and retention in tumor tissues, thus enhancing the targeting of radiotherapy.^45^ EFolic acid-conjugated palladium-based nanoparticles achieve selective accumulation by targeting overexpressed folate receptors in breast cancer cells. This reduces the off-target toxicity and expands the precision of radiopharmaceutical therapy.^46^ Different palladium-based radiotherapy materials have unique focuses and limitations. Bimetallic alloys offer better sensitization efficiency than single components but require more complex synthesis. The targeted mechanism for hypoxia regulation of PdTe depends on intratumoral H_2_O_2_. Its effect on tumors with low H_2_O_2_ was obviously restricted. FePd@CNTs’ ability to penetrate biological barriers is suitable for solid tumors. However, the long-term biocompatibility of carbon nanotubes needs further verification. Folic acid targeting improves specificity but is susceptible to receptor differences due to tumor heterogeneity. Some cancer cells may escape. Overall, these strategies upgrade from simple radiosensitization to microenvironment regulation plus targeted delivery. The core logic is to optimize radiotherapy efficacy and reduce side effects by utilizing the properties of palladium nanomaterials. Most studies, however, remain at the in vitro or small animal model stage, and there is insufficient pharmacokinetic and long-term safety data from large animal experiments. Additionally, large-scale synthesis and clinical transformation of some materials present challenges. In the future, it is necessary to strengthen the matching of material structure, mechanism of action, and tumor type. Preclinical large-sample verification and accelerated transformation from basic research to clinical applications are also needed.

### Chemotherapy

The application of palladium nanomaterials in the field of chemotherapy focuses on two core directions: precise drug delivery and direct antitumor effects, providing innovative solutions with both targeted and safe approaches for cancer treatment. As efficient drug carriers, Pd NPs can achieve explicit delivery and controlled release of chemotherapeutic drugs. The bimetallic gold-core palladium dendritic shell nanoparticles (Au@PdNDs) developed by Oladipo and colleagues can stably load doxorubicin (DOX) and achieve pH-responsive release, offering a new path for multistimuli-responsive drug delivery.^18^ The palladium nanoparticle-cisplatin (CisPt) nanodelivery system, synthesized by Bellissima and colleagues through a green redox method, exhibits dual-modal therapeutic potential. And its antitumor effect has been verified by in vitro experiments.^47^ To address treatment challenges such as cancer-related anemia and tumor microenvironment heterogeneity, Zhu et al. constructed an intelligent nanosystem based on palladium nanoplates (PdPL) to deliver tocilizumab (TCZ), which corrects anemia and inhibits tumor progression by blocking the IL-6/IL-6R signal.^48^ Safarkhani and colleagues modified palladium (PdL(2)) ligands on carbon-based nanocarriers, achieving efficient targeted drug delivery to breast cancer cells with low toxicity to normal cells.^49^ The resveratrol-functionalized palladium nanoparticles (Res–PdNPs) synthesized by Thipe et al. using green nanotechnology can target and regulate the NF−κB signaling pathway, induce the conversion of M2-type macrophages to M1-type, and exhibit selective toxicity in prostate tumor treatment, which is superior to traditional chemotherapeutic drugs such as cisplatin.^50^ In terms of directly exerting chemotherapeutic effects, palladium-based compounds and nanosystems have shown significant abilities to resist proliferation, induce DNA damage, and promote apoptosis. To address the problem of platinum drug resistance in triple-negative breast cancer (TNBC), the trinuclear chelate Pd(3)­Spd(2) formed by palladium­(II) and spermine has significant antiproliferative activity against both cisplatin-sensitive and resistant TNBC cells, with good selectivity for normal breast cells.^51^ The biomineralized nanosystem CaCO3@Pd@C, developed by Liang et al., regulates the tumor microenvironment and exhibits good biocompatibility, as well as a rapid induction of tumor cell apoptosis in both cellular and animal experiments.^52^ Due to the aggregation of chemically synthesized nanoparticles under physiological conditions, their application in biomedicine is limited. Feng et al. successfully prepared palladium (Pd)/zinc oxide (ZnO) bimetallic nanocomposites (CS Pd/ZnO NCs) using Crocus sativus extract. This material can induce cell cycle arrest and apoptosis in cervical cancer, serving as an effective chemotherapy strategy for cervical cancer patients.^53^ Gunes et al. found that, compared with polyethyleneimine-functionalized nitrogen-doped graphene quantum dots (PEI-N-GQDs, which are tiny particles modified to enhance delivery in cells), palladium nanocomposites (PdNPs/PEI-N-GQDs, which are Pd NPs combined with these functionalized quantum dots) have a smaller particle size and a specific shape. This enhances the ability of the particles to enter cells, which in turn increases their potential to kill ovarian cancer cells by triggering apoptosis. This study suggests that PdNPs/PEI-N-GQDs could be a promising new approach for ovarian cancer chemotherapy.^54^

### Novel Therapy

#### Sonodynamic Therapy

Redox nanoenzymes show great potential in disrupting cellular homeostasis in cancer treatment; nevertheless, their different active dysfunctions compete, often limited by the intricate TME. Since Pd nanocrystals can precisely regulate enzyme-like activity by adjusting the exposed crystal facets, noble metal nanoalloys can improve enzyme-like activity by promoting electron transmission and strengthening active sites. This study designed a PdCu­(x)@LDH bimetallic nanoalloy, demonstrating optimized enzyme activity and efficient singlet oxygen generation capacity. Its unique surface-dependent polyenzyme-like activity is further enhanced under ultrasound stimulation, achieving mitochondrial dysfunction and efficient alloy nanoenzyme-mediated cancer treatment. This opens up a new research path of sonodynamic therapy (Figure 3A).^19^

**Figure 3:**
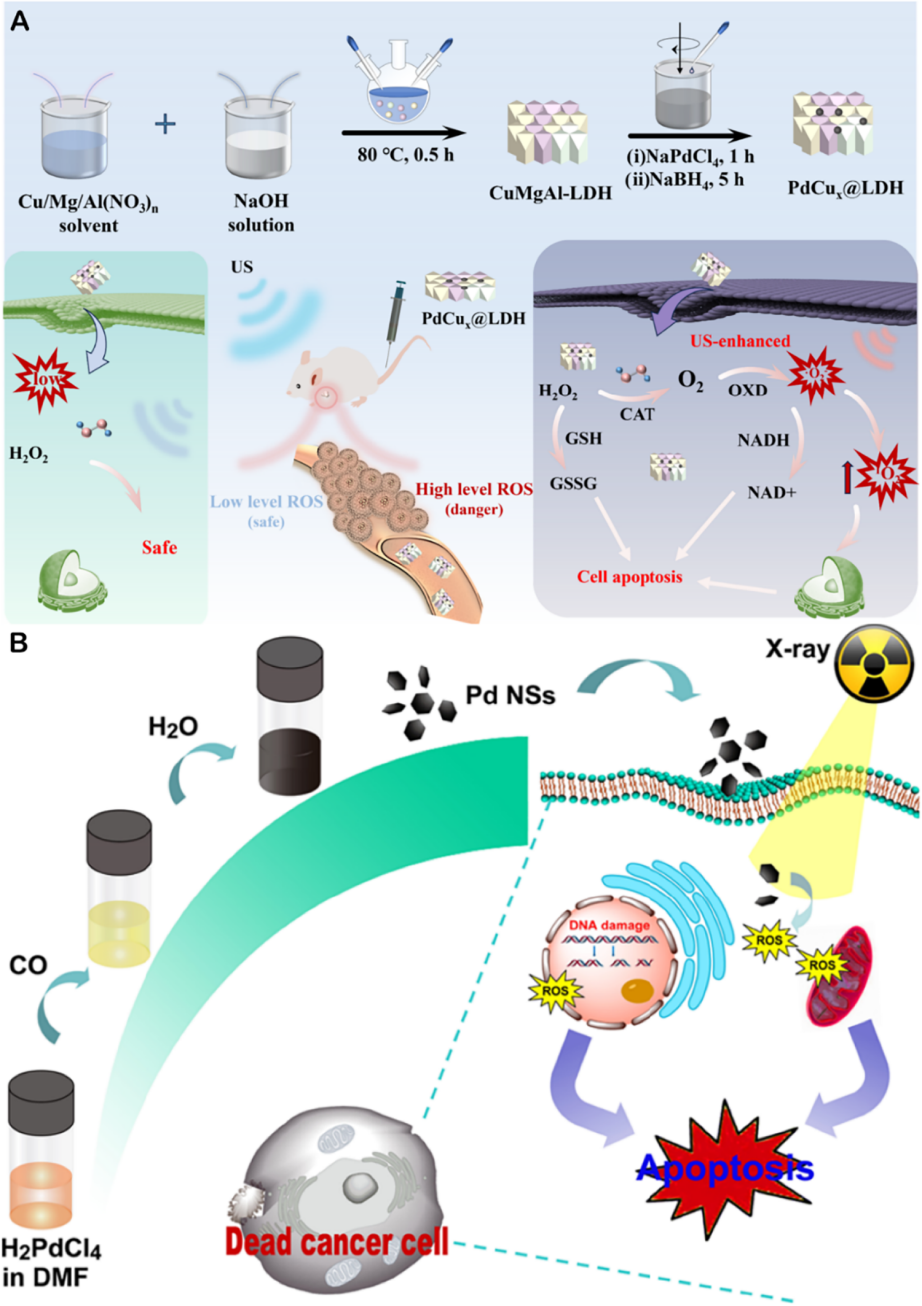
(A) Schematic diagram for the preparation of the PdCux@LDH Alloy nanozymes and the proposed antitumor mechanism, reprinted with permission from ref 19. Copyright 2024, American Chemical Society. (B) Schematic showing the synthetic route of Pd NSs and their application in cancer cell radiosensitization, reprinted with permission from ref 55. Copyright 2020, American Chemical Society.

#### Magnetic Hyperthermia Therapy

Magnetic hyperthermia is a technique that utilized a magnetic field to generate heat for therapeutic purposes. Magnetic hyperthermia mainly utilizes electromagnetic waves to produce heat, causing magnetic nanoparticles to vibrate and aggregate under an alternating magnetic field, thereby generating heat. This heat can act on human tissues to achieve a therapeutic effect. Researchers found that Pd/Fe-oxide magnetic nanoparticles composed of a nonmagnetic palladium core and a magnetic iron oxide shell showed excellent magnetic hyperthermia/thermal ablation potential under clinically safe conditions of 346 kHz and 19.1 mT, with minimal eddy current effects and maximum specific loss power, providing a new efficient nanomaterial choice for cancer magnetic hyperthermia therapy.^56^

#### Piezoelectric Catalytic Therapy

Piezoelectric catalytic tumor treatment is an emerging ROS production therapy that depends on piezoelectric polarization under the irradiation of ultrasound (US). Improving ROS creation is the main goal to improve treatment efficiency. Deng et al. found that defect engineering-optimized oxygen vacancy-rich integrated barium titanate (BTO) nanoparticles enhanced ROS production efficiency and palladium’s dual enzyme-mimetic characteristics. This nanoparticle achieved efficient piezoelectric catalytic treatment of mouse breast cancer, showing this material’s important application potential in tumor treatment.^20^

#### Bioorthogonal Catalysis Therapy

Bioorthogonal catalysis offers a useful device for non-natural chemical reactions in living systems to analyze intricate processes in cells. However, the ability to precisely control cell functions is limited due to the deficiency of bioorthogon catalysts with cell selectivity. Chen and colleagues reported Pd NPs (Pd@UiO-66) deposited on the metal–organic framework UiO-66 as an efficient intracellular bioorthogonal catalyst, and by introducing cancer cell-targeting aptamer AS1411, significantly improved its catalytic efficiency in cancer cells, achieving precise regulation of specific protein functions, providing a new chemical biology strategy for the spatiotemporal control of protein functions in therapeutic applications.^57^ Tang et al. developed a high-performance nanocomposite based on ultrafine Pd NPs using a molecular cage encapsulation strategy. By combining GOx and hyaluronic acid (HA) modified with AS1411 aptamer, the catalytic activity was significantly improved. This nanocomposite can selectively synthesize drugs upon the activation of acidic pH and hyaluronidase, which is overexpressed in the tumor environment. It also has the functions of promoting the generation of ROS and depleting intracellular glutathione (GSH), thus enhancing the therapeutic effect on tumors.^58^ Besides, Gurunathan et al. found that oxidative stress is one of the most critical mechanisms underlying the cytotoxic effect of Pd NPs on ovarian cancer cells. Additionally, mitochondrial dysfunction and lipid peroxidation are significant contributors to apoptosis in ovarian cancer cells induced by Pd NPs. This study suggests that Pd NPs may serve as a novel therapeutic strategy for treating ovarian cancer.^59^

### Combination Therapy

Given the limitation of PDT in shallow light penetration, it is often used in combination with PTT, and the introduction of Pd NPs further enhances the synergistic effect between the two: Researchers synthesized a smart hydrogel Pd/DOX@hydrogel loaded with doxorubicin and Pd NPs, which not only achieves synergistic tumor killing through PTT and PDT but also acts as a temporary bionic skin to promote wound healing.^6^

In addition, Zhang et al. further developed a nanobacterial biohybrid EcN–QS–iLux@Pd@MC540. Through biomineralization of engineered probiotics and Pd NPs, as well as loading of the photosensitizer MC540, effective self-driven PDT and PTT were achieved, resulting in complete tumor suppression in a 4T1 tumor mouse model with no obvious systemic toxicity.^60^ Beyond the combination of PDT and PTT, the synergistic scheme of PTT and CDT has also made breakthroughs: The MnO_2_@Pd@PPy/GO nanocomposite, with its excellent photothermal conversion performance, pH-responsive enzymatic catalytic activity, and enhanced MRI performance, realizes MRI-guided precise synergistic therapy of the two, becoming a highly effective and low-side-effect cancer “nanotherapeutic agent”.^15^ The graphene oxide nanozyme rGO-Pd reported by Sun et al. further enhances ROS production under the dual effects of tumor microenvironment acidity and photothermal effect by virtue of enhanced peroxidase-like activity and near-infrared light responsiveness, providing a new direction for this combined strategy.^61^ Meanwhile, to address the problems of hydrogen delivery in hydrogen therapy, as well as hypoxia and low electron–hole separation efficiency in PDT, Wang and colleagues developed a yolk–shell structured nanoplatform PdH@MnO_2_·Ce6@HA (PHMCH), which innovatively integrates the advantages of stable hydrogen storage and release, PTT, and enhanced PDT, achieving H_2_-mediated gas therapy combined with the synergistic antitumor effect of the latter two.^62^

In the field of combining PTT with chemotherapy, Wang and colleagues developed a visual photothermal-controlled drug release nanosystem (VPNS) to address the resistance of pancreatic ductal adenocarcinoma (PDAC) to gemcitabine (GEM), successfully inhibiting PDAC cell proliferation through dual-mode therapy.^63^ Sathiyaseelan et al. synthesized folic acid (FA)- and chitosan (CS)-coated quercetin (R)-palladium nanoclusters (Pd-NCs), which can effectively inhibit the proliferation of breast cancer MDA-MB231 cells under near-infrared light irradiation.^64^ The transferrin (Tf)-conjugated nanoplatform (Tf-PPPs) developed by Nguyen combines porous Pd NPs with paclitaxel (PTX) to achieve chemo-photochemical combined therapy on MCF-7 tumor cells under near-infrared light irradiation (Nguyen, Soe et al.). Foti et al. provide a new strategy for photothermal combined chemotherapy in prostate cancer. They synthesized polyvinylpyrrolidone (PVP)-coated gold, silver, and Pd NPs via a green redox method, which, with their photothermal and antiangiogenic properties.^65^

In the combination of radiotherapy and chemotherapy, the radiosensitizing potential of Pd NPs has been fully explored: Jiang et al. found that flake-like Pd-NSs, as sensitizers, combined with X-rays can promote cell apoptosis by inducing DNA double-strand breaks and ROS production, demonstrating their application value in the combination of radiotherapy and chemotherapy (Figure 3B).^55^

Besides, thermal ablation therapy combined with brachytherapy is also a promising combination therapy. Current early stage breast cancer treatments include surgical tumor removal and fractionated dose radiation therapy. Van Oossanen et al. developed radioactive magnetic nanoparticles (RMNPs) containing palladium-103. RMNP seeds deliver a dose distribution that is comparable to current commercial brachytherapy seeds, accompanied by a decrease in dose anisotropy. The surgical placement of the RMNP seeds is easier than that of commercial brachytherapy seeds. This novel strategy combines thermal ablation therapy and brachytherapy for breast cancer, potentially improving patient prognosis.^66^

In terms of enhancing immunotherapy, Pd-mediated combination schemes also perform prominently: The nanosystem (Pd-Dox@TGMs NPs) developed by Wen and colleagues effectively triggers immunogenic cell death (ICD) through the combination of PTT and chemotherapy, thereby enhancing the PD-L1 blocking effect and promoting cytotoxic T lymphocyte infiltration (Figure 4A,B).^8^ The multifunctional copper-based nanocomposite (RCL@Pd@CuZ), further developed by Li et al., effectively improves radiotherapy sensitivity and enhances ICD by improving tumor microenvironment hypoxia and consuming GSH.^68^ Ming and colleagues used Pd-NSs as carriers for the immune adjuvant CpG-ODNs (Figure 4C), and the combination with PTT significantly improved the tumor treatment efficacy.^67^ Sungu et al. found that Zn–Ni–FeO and Pd NPs can induce macrophage polarization to the M1 type, and the derived exosomes (PdNPs-Exo) combined with 5-fluorouracil can further enhance the chemotherapy effect on breast cancer.^69^ The M13@Pd/NLG gel system, developed by Dong et al., integrates photothermal Pd NPs and NLG919. It can reshape the tumor microenvironment, induce ICD, and improve the response rate of breast cancer treatment.^70^ Xie et al. prepared functional lipid nanoparticles (FPS-LNPs) encapsulating iron–palladium nanozymes (FePd-NZ) and shikonin (SKN). FPS-LNPs can enhance necroptosis of tumor cells by generating additional intracellular reactive oxygen species, achieving significant inhibition of tumor growth. In addition, tumor cells undergoing necroptosis can release damage-associated molecular patterns (DAMPs) and antigens, thereby triggering the immune response of CD8^+^ T cells. This study presents a novel strategy for combining CDT with immunotherapy for treating tumor patients (Figure 5A,B).^71^ Feng et al. constructed a bioorthogonal nanoplatform (HBPdC), based on CRISPR/Cas9 gene-edited Pd nanomaterials and a HA surface layer. This HBPdC could enhance the therapeutic effect of cancer treatment by promoting macrophage phagocytosis and inducing CDT.^72^ Photoimmunotherapy has the characteristics of spatiotemporal precision and noninvasiveness, and it is also a combined treatment strategy. Feng et al. provided a new photoimmunotherapy combination strategy for highly malignant TNBC. They prepared a rod-shaped plasmonic gold–palladium heterostructure (Au–Pd–HS), which, combined with NIR light irradiation, utilizes its strong photothermal and photodynamic effects to trigger ICD and enhance immune response. Meanwhile, combined with anti-PD-L1 therapy, it reverses the immunosuppressive microenvironment and improves the therapeutic effect in TNBC patients.^73^

**Figure 4:**
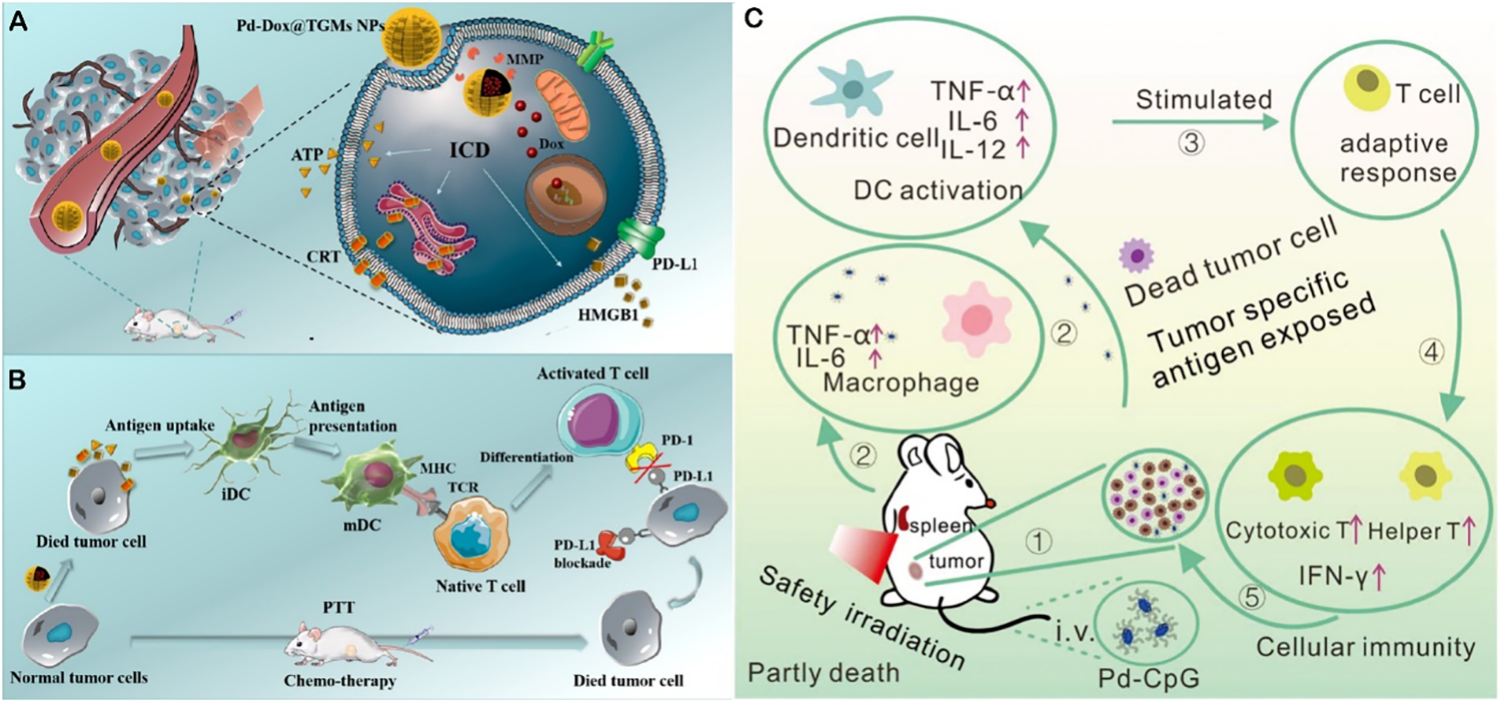
(A) Pd-Dox@TGMs NPs mediated ICD process via chemo- and PTT after reaching the tumor Site through EPR Effect, reprinted with permission from ref 8. Copyright 2019, American Chemical Society. (B) Immune activation process under Pd-Dox@TGMs NPs treatment, reprinted with permission from ref 8. Copyright 2019, American Chemical Society. (C) Possible mechanism of Pd (5)-CpG (PS) facilitated cancer killing that combined immunotherapy and photothermal treatment, reprinted with permission from ref 67. Copyright 2020, Royal Society of Chemistry.

**Figure 5:**
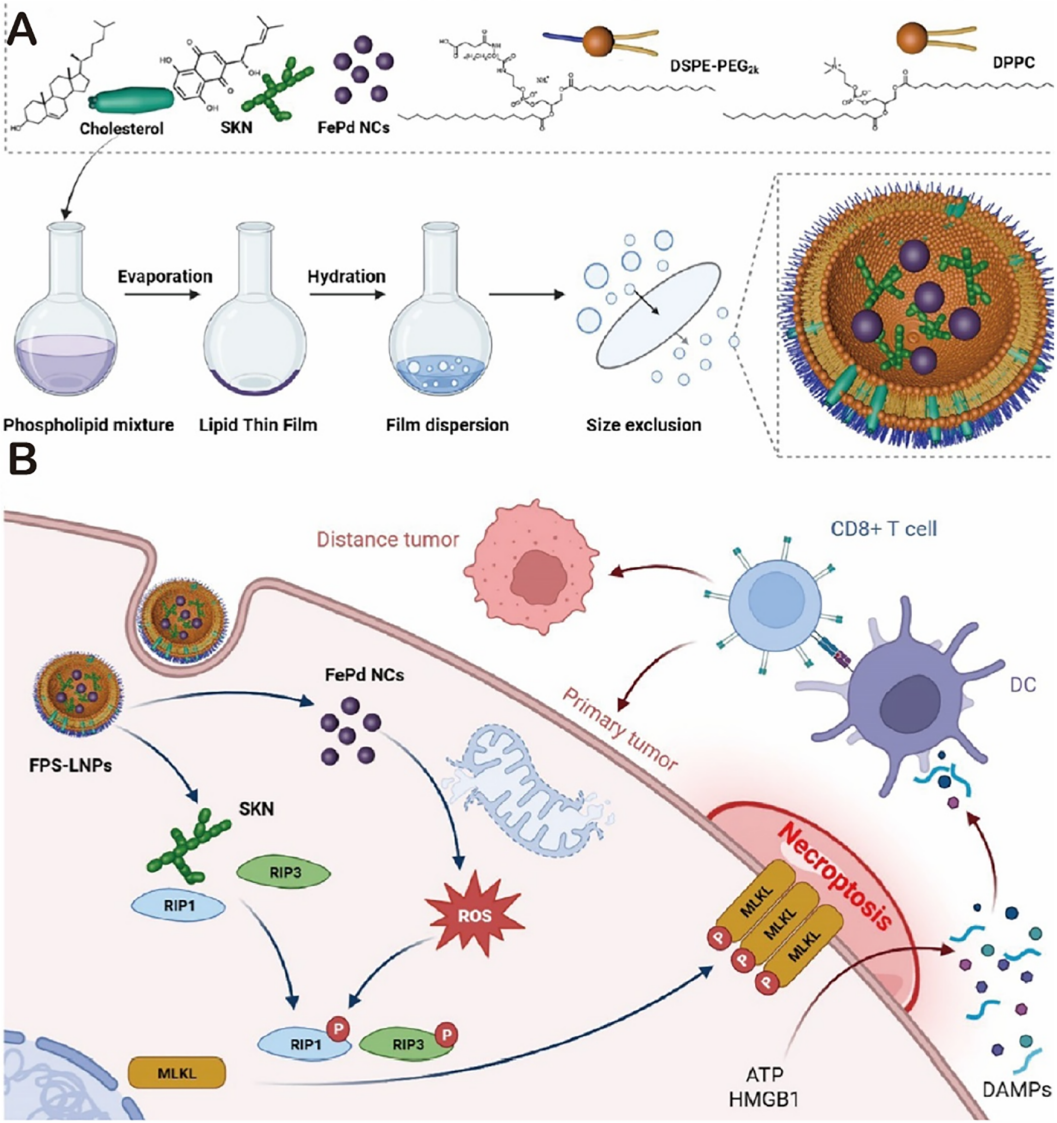
(A) Schematic illustration of the synthetic procedures via the thin-film hydration strategy to prepare both FePdNZ- and SKN-encapsulated functional lipid nanoparticles (FPS-LNPs) reprinted with permission from ref 71. Copyright 2024, American Chemical Society. (B) Antitumor mechanisms of FPS-LNPs via ROS-enhanced SKN-mediated necroptosis induction and CD8^+^ T cell immune response activation, reprinted with permission from ref 71. Copyright 2024, American Chemical Society.

## Challenges and Prospects

Compared to other noble metal nanomaterials, such as gold, silver, and platinum, Pd-NMs offer significant advantages. In addition to excellent photothermal conversion efficiency, stability, and biocompatibility (Table 3), Pd-NMs also possess outstanding electrocatalytic performance in oxygen reduction reactions (ORR) (which can regulate the redox state of the tumor microenvironment), strong hydrogen dissociation ability (suitable for hydrogen concentration sensing to monitor treatment responses), and room for optimization through modification with nonmetallic atoms (such as H, B, C; for example, carbon doping enhances conductivity). Furthermore, when Pd-NMs form bimetallic or trimetallic materials with Au or Ag, they can produce a synergistic catalytic effect through electron transfer, such as improving the efficiency of ROS generation in CDT. However, Pd-NMs also have shortcomings: their pure-state catalytic performance is difficult to meet clinical needs and requires optimization through alloying or surface modification, whereas the pure-state activities of gold and platinum already support some single-function therapies. In addition, compared with gold (Au) and silver (Ag), Pd-NMs have weaker optical properties and poorer plasmonic effects. This characteristic puts Pd-NMs at a relative disadvantage in two key application scenarios: one is SERS imaging, and the other is the light absorption efficiency in PDT. Meanwhile, the performance of Pd-NMs is also easily affected by electron density. Specifically, as the particle size increases, the distribution of surface electron clouds changes, leading to a significant decrease in catalytic activity.^74^

**Table 3:** Analysis of the Advantages and Disadvantages of Palladium, Gold, Platinum, and Silver Nanomaterials^a^

category	advantages	disadvantages	citation
palladium nanomaterials	(1) excellent electrocatalytic performance (Oxygen Reduction Reaction, ORR)	(1) the catalytic performance of pure palladium nanomaterials is usually unsatisfactory	^74^
	(2) strong hydrogen dissociation ability, suitable for hydrogen sensing	(2) the optical properties are weaker than those of Au and Ag (with poor plasmonic effects)	
	(3) performance can be improved through modification with nonmetallic atoms (H, B, C, etc.)	(3) it is easily affected by the electron density distribution (for example, the activity decreases when the particle size increases)	
	(4) it has a synergistic catalytic effect when forming bimetallic/trimetallic nanomaterials with Au and Ag		
gold nanomaterials	(1) high biocompatibility and chemical inertness	(1) the catalytic efficiency may be lower than that of palladium-based bimetallic materials (for example, the efficiency of Au–Pd in catalyzing the reduction of 4-NP is the lowest)	^75,76^
	(2) the surface is easy to functionalize, suitable for biosensing (such as CRISPR-Cas detection)		
	(3) strong plasmonic effect (high extinction coefficient)	(2) the cost is relatively high	
	(4) antibacterial properties can be adjusted (depending on size/morphology)		
platinum nanomaterials	(1) oxidase/peroxidase mimetic activity (inducing ROS, antibacterial)	(1) bacteria may develop resistance to it	^77^
	(2) When forming an alloy with palladium (Pd) (such as PdPt), it can enhance the catalytic duration	(2) there is relatively little research (compared to Au and Pd)	
silver nanomaterials	(1) excellent antibacterial performance	(1) easy oxidation leads to poor stability	^76,78^
	(2) high catalytic efficiency when forming bimetallic materials with Pd or Au (for example, Ag–Pd is optimal for catalyzing the reduction of 4-NP)	(2) the risk of biological toxicity is relatively high	

a Abbreviation: ORR, Oxygen Reduction Reaction; ROS, Reactive Oxygen Species; 4-NP, 4-nitrophenol.

Based on recent studies, we found that Pd-NMs can serve as imaging contrast agents, detectors of CTCs, and tumor biomarkers such as CEA and PSA, aiding in tumor diagnosis. Additionally, there have been numerous studies on the application of Pd-NMs in PTT, PDT, CDT, chemotherapy, radiotherapy, and combination therapies, but their clinical translation and application still face multiple challenges.

In terms of clinical translation, most Pd-NMs research remains at the stage of in vitro cell and animal models, with only a few formulations entering Phase I clinical trials, and no Phase II/III diagnostic or therapeutic formulations available.

There are three main core obstacles: first, there are differences between preclinical models and the human tumor microenvironment, making it difficult to reproduce therapeutic effects. For example, their immune responses, vascular permeability, and metabolic pathways are all different; second, there is a lack of long-term safety data. Aspects such as chronic toxicity, cumulative effects of immunogenicity, and specific impacts on organ function are currently unclear. Third, it is difficult to meet the regulatory requirements for nanomedicines in terms of standardized quality control during the synthesis process and batch reproducibility.

Furthermore, the unclear mechanism of synergistic toxicity in Pd-NMs combination therapies (e.g., PTT-induced oxidative stress exacerbating chemotherapy-induced myelosuppression) hinders the development of safety protocols. Biosafety is another core bottleneck: Pd-NMs affect the immune system by interfering with immune cell functions and inducing oxidative stress. For example, polystyrene-palladium nanoparticles (PS-Pd NPs) can interact with 37 subtypes of human blood immune cells, leading to immune regulation disorders.^79^ However, surface modifications (such as carboxymethyl cellulose (CMC) and PGA) can reduce their immunogenicity.^80^ Although anisotropic Pd-NMs have high NIR absorption efficiency, they may trigger stronger immune responses, and the underlying mechanism remains to be explored. In terms of toxicology, their toxicity is organ-specific, concentration-dependent, and morphology-dependent. Long-term exposure induces pulmonary oxidative stress (abnormal superoxide dismutase (SOD) and catalase (CAT) activities, GSH depletion, and increased lipid peroxidation (LPO)) and triggers inflammation.^81^ The highest accumulation occurs in the kidneys (>10 μg/g), and high doses (≥400 μg/mL) can induce apoptosis and ROS bursts (Aarzoo, Naqvi et al.). Metabolic clearance is influenced by particle size, surface modification, and administration route. Serum palladium exhibits biphasic kinetics (with half-lives of 20.7 h for the bound state and 35.5 h for the free state).^82^ The kidneys and liver are the main clearance organs. Ultrasmall Pd-NMs (e.g., 3.2 nm Pd@insulin) can be rapidly cleared via the renal-urinary pathway,^83^ while larger particles tend to accumulate in tissues.^84^ Additionally, there are still technical optimization challenges: targeted delivery requires balancing the targeting ability of surface modifications with the risk of nontarget organ accumulation; the mechanism of synergistic toxicity in combination therapies is unclear; Pd-NMs-based detectors in the field of CTC detection are scarce and suffer from interference from blood matrices, making it difficult to identify EpCAM-negative CTCs.^85^ In terms of industrialization, the cost of palladium extraction and synthesis is high, existing methods (such as chemical reduction) have low yields, poor batch reproducibility, and are difficult to scale up, and there is no unified standard for synthesis processes and quality control, which restricts clinical application.

In the future, breakthroughs in these challenges need to be made in multiple directions: To promote clinical translation, it is necessary to develop patient-derived xenograft (PDX) and tumor organoid models to reduce the gap between preclinical and clinical settings, conduct multicenter long-term clinical trials to accumulate safety and efficacy data, and collaborate with regulatory agencies to establish quality standards for Pd-NMs (such as limits on palladium ion release and particle size ranges).

To enhance the biosafety and targeting accuracy of Pd-NMs, their design and research can be optimized from multiple dimensions: on the one hand, by regulating the particle size within the range of 5–10 nm to enhance renal clearance capacity, reducing accumulation in nontarget organs in the body; at the same time, using PEG for surface modification to reduce immunogenicity and minimize interference with the immune system; in addition, it is necessary to deeply analyze the correlation mechanism between material morphology and immune response to provide theoretical support for the development of Pd-NMs with low immune risk. To improve targeting efficiency, dual-targeting ligands (such as the combination of RGD and transferrin) can be developed, or pH-sensitive surface modifications can be employed. By leveraging the characteristics of the tumor microenvironment, specific accumulation of the materials at the tumor site can be achieved, further enhancing the targeted binding ability to tumor tissues and reducing damage to normal tissues. Innovation in diagnostic and therapeutic strategies should focus on the synergistic effect mechanisms of Pd-NMs, such as PTT-induced ICD, constructing pH-sensitive carriers for on-demand drug release, developing dual-signal detectors (photothermal + SERS) to improve CTC detection sensitivity and identify multiple CTC subtypes, and utilizing bioorthogonal catalysis for in situ prodrug activation or tumor labeling. In interdisciplinary applications, Pd-reduced graphene oxide (Pd-RGO) nanocomposites (which upregulate GAP-43 and MAP2 to protect brain tissue)^86^ can be used to repair neurotoxicity induced by chemotherapy/radiotherapy, explore their synergistic mechanisms in tumor microenvironment regulation and neuroprotection, and develop ″diagnosis-treatment-repair″ platforms. For industrialization challenges, Pd–Cu and Pd–Ag alloys can be developed to reduce palladium usage, green synthesis methods, such as plant extract reduction, can be promoted, flow chemistry processes can be optimized to improve yield and reproducibility, and good manufacturing practice (GMP) large-scale manufacturing processes can be established. Overall, Pd-NMs show significant potential in the field of tumor diagnosis and treatment. With the continuous advancement of nanotechnology, through precise structural design of Pd-NMs, in-depth elucidation of their mechanism of action, integrated application of interdisciplinary technologies, and establishment of standardized production processes, such nanomaterials are expected to gradually break through the current transformation bottlenecks, achieve the leap from laboratory research to clinical application, and ultimately provide new solutions and treatment options for the field of precise cancer treatment.

## Conclusions

Pd-NMs have a vast potential for application in tumor diagnosis and treatment. Pd-NMs’ unique properties, including excellent catalytic activity, good biocompatibility, and photothermal effect, give them significant advantages in imaging, tumor cell detection, biomarker detection, PTT, PDT, CDT, chemotherapy, radiotherapy, and combination therapy. With the advancement of nanotechnology and the development of new Pd-NMs, Pd-NMs have brought breakthroughs in tumor diagnosis and treatment. However, to achieve their widespread application in clinical treatment, challenges such as how to improve the targeting of tumor tissues, how to ensure long-term biosafety, and how to reduce costs and achieve large-scale production need to be overcome. Future research will focus on improving the therapeutic effect of Pd-NMs on tumor cells, developing novel treatment strategies, studying their long-term biosafety in the human body, and promoting their clinical transformation and application. In conclusion, Pd-NMs have great potential and advantages in tumor diagnosis and treatment, but there are some challenges and issues to be addressed. With the rapid development and innovation of nanotechnology, we look forward to more breakthrough progress of Pd-NMs making a greater contribution to tumor diagnosis, therapy, and human health.

## Abbreviation

Au: gold
aM: attomolar, 1 aM = 10^–18^ mol/L
CA-242: carbohydrate antigen 242
CDT: chemodynamic therapy
CEA: carcinoembryonic antigen
COP: covalent organic polymers
CS: chitosan
CT: computed tomography
DOX: doxorubicin
ECL: electrochemiluminescence
EPR: enhanced permeability and retention
GEM: gemcitabine
GSH: glutathione
GSS: glutathione synthetase
HER2: human epidermal growth factor receptor 2
ICD: immunogenic cell death
LFA: lateral flow analysis
LSCC: laryngeal squamous cell carcinoma
MRI: magnetic resonance imaging
mL: milliliter
NAA: *N*-acetylaspartate
NIR: near-infrared
NSCLC: non-small cell lung cancer
PA: photoacoustic
PD-L1: programmed cell death ligand 1
Pd-NMs: palladium-based nanomaterials
Pd NPs: palladium nanoparticles
Pd NSs: palladium nanosheets
PDT: photodynamic therapy
PEC: photoelectrochemical
PET: positron emission tomography
PPTT: plasmonic photothermal therapy
PS: photosensitizers
PSA: prostate-specific antigen
PTT: photothermal therapy
ROS: reactive oxygen species
SDT: sonodynamic therapy
SPECT: single photon emission computed tomography
TAMs: tumor-associated macrophages
TME: tumor microenvironment
TNBC: triple-negative breast cancer
XFI: X-ray fluorescence imaging
